# Expression of Novel Opsins and Intrinsic Light Responses in the Mammalian Retinal Ganglion Cell Line RGC-5. Presence of OPN5 in the Rat Retina

**DOI:** 10.1371/journal.pone.0026417

**Published:** 2011-10-17

**Authors:** Paula S. Nieto, Diego J. Valdez, Victoria A. Acosta-Rodríguez, Mario E. Guido

**Affiliations:** CIQUIBIC (CONICET)- Departamento de Química Biológica, Facultad de Ciencias Químicas, Universidad Nacional de Córdoba, Córdoba, Argentina; University of Houston, United States of America

## Abstract

The vertebrate retina is known to contain three classes of photoreceptor cells: cones and rods responsible for vision, and intrinsically photoresponsive retinal ganglion cells (RGCs) involved in diverse non-visual functions such as photic entrainment of daily rhythms and pupillary light responses. In this paper we investigated the potential intrinsic photoresponsiveness of the rat RGC line, RGC-5, by testing for the presence of visual and non-visual opsins and assessing expression of the immediate-early gene protein c-Fos and changes in intracellular Ca^2+^mobilization in response to brief light pulses. Cultured RGC-5 cells express a number of photopigment mRNAs such as retinal G protein coupled receptor (RGR), encephalopsin/panopsin (*Opn3)*, neuropsin (*Opn5)* and cone opsin (*Opn1mw*) but not melanopsin (*Opn4)* or rhodopsin. Opn5 immunoreactivity was observed in RGC-5 cells and in the inner retina of rat, mainly localized in the ganglion cell layer (GCL). Furthermore, white light pulses of different intensities and durations elicited changes both in intracellular Ca^2+^ levels and in the induction of c-Fos protein in RGC-5 cell cultures. The results demonstrate that RGC-5 cells expressing diverse putative functional photopigments display intrinsic photosensitivity which accounts for the photic induction of c-Fos protein and changes in intracellular Ca^2+^ mobilization. The presence of Opn5 in the GCL of the rat retina suggests the existence of a novel type of photoreceptor cell.

## Introduction

Retinal ganglion cells (RGCs) play a key role in the circadian system of all vertebrates, being responsible for synchronizing central pacemakers to the environmental illumination conditions that coordinate the temporal organization of behavior and physiology. RGCs send visual and photic information to the brain through the axons forming the optic nerve and projecting to areas in the central nervous system involved in image- and non-image-forming tasks [1]–[5]. In vertebrates, a subset of intrinsically photosensitive retinal ganglion cells (ipRGCs) expressing the photopigment melanopsin (Opn4) [6]–[8] are responsible for transducing information about ambient lighting conditions to brain areas involved in non-image-forming tasks (entrainment of the circadian clock, pupillary light reflexes and suppression of melatonin synthesis) [1], [2], [7], [9]–[11]. These ipRGCs may have evolved from a common ancestor with rhabdomeric photoreceptors of invertebrates [6], [12]–[19]. Our recent observations support the idea that the phototransduction cascade operating in primary cultures of chicken RGCs is closely related to that taking place in rhabdomeric cells, involving a phosphoinositide cascade [18], [20]. In addition, cells of the inner retina may express other photopigments and photoisomerases such as encephalopsin/panopsin (OPN3), neuropsin (OPN5), peropsin, retinal G protein coupled receptor (RGR), vertebrate ancient (VA) opsin and cone opsins [4], [21]–[28]. Though their exact function in the retina is unknown, these photopigments may cooperate with classical opsins in the process of photon capture and chromophore regeneration, or be directly involved in tasks requiring precise photic responsiveness.

A clonal rat retinal cell line named RGC-5 displays RGC characteristics based on expression of specific markers such as Thy-1, Brn-3c, Neuritin, NMDA and GABAb receptors, sensitivity to glutamate excitotoxicity and neurotrophin withdrawal [29]–[31]. These cells constitute a widely used model for studying physiological and pathophysiological processes in retinal cells. We recently demonstrated that proliferating RGC-5 cells express different clock genes such as *Per1, Clock* and *Bmal1*, while serum stimulation induced the expression of the immediate-early gene (IEG) proteins c-Fos and PER1 [29]. IEGs encode for, among others, the inducible transcription factors of the Fos and Jun families; they are rapid and transiently induced in response to diverse physiological stimuli, acting as cellular messengers in the coupling of extracellular signals to long-term cellular changes [32]. In the retina, light stimulation selectively induces IEG expression in RGCs of mammals and birds [33]–[37] and in neurons of the central pacemaker, the suprachiasmatic nucleus (SCN) [38]–[42].

In the present work we investigated the expression of different classical and non-visual opsins and potential intrinsic light responsiveness in RGC-5 cells. To this end, we first examined the mRNA expression of RGR, *Opn3*, *Opn4*, *Opn5* and classical opsins such as rhodopsin and cone opsin *Opn1mw* in these cells. We furthermore assessed the presence of Opn5 protein in the retina of rat and in RGC-5 cells and examined the differential RGC-5 responses to light exposure related to inducing IEG expression and/or causing changes in the mobilization of intracellular Ca^2+^.

## Results

### Expression of Classical and Non-visual Opsins in RGC-5 Cultures

**Figure 1:**
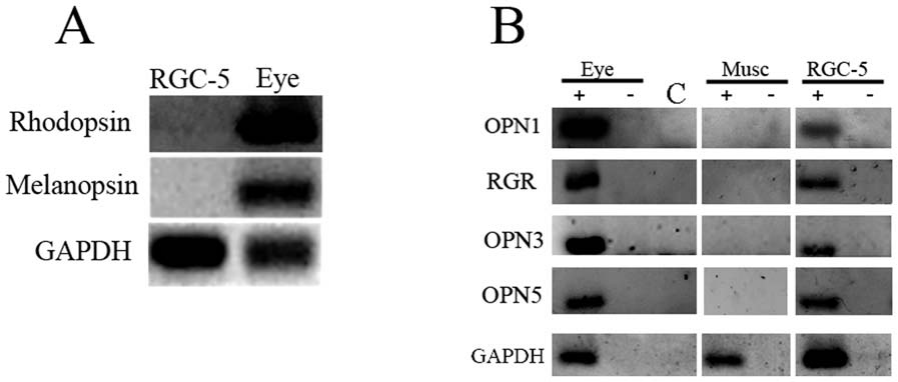
Expression of Visual and Non-visual mRNA Photopigments in RGC-5 cells. **A:** Assessment of *Rhodopsin, Opn4* and GAPDH mRNAs by RT-PCR in RGC-5 cells and whole rat eye (Eye). **B:** Assessment of *Opn1mw, RGR, Opn3, Opn5* and GAPDH mRNAs by RT-PCR in whole rat eye (Eye), muscle (Musc) and RGC- 5 cells, in the presence (+) or absence (−) of reverse transcriptase, indicating that the amplification observed is not due to genomic-DNA residues. Control (C) is the no template control.

**Figure 2 pone-0026417-g002:**
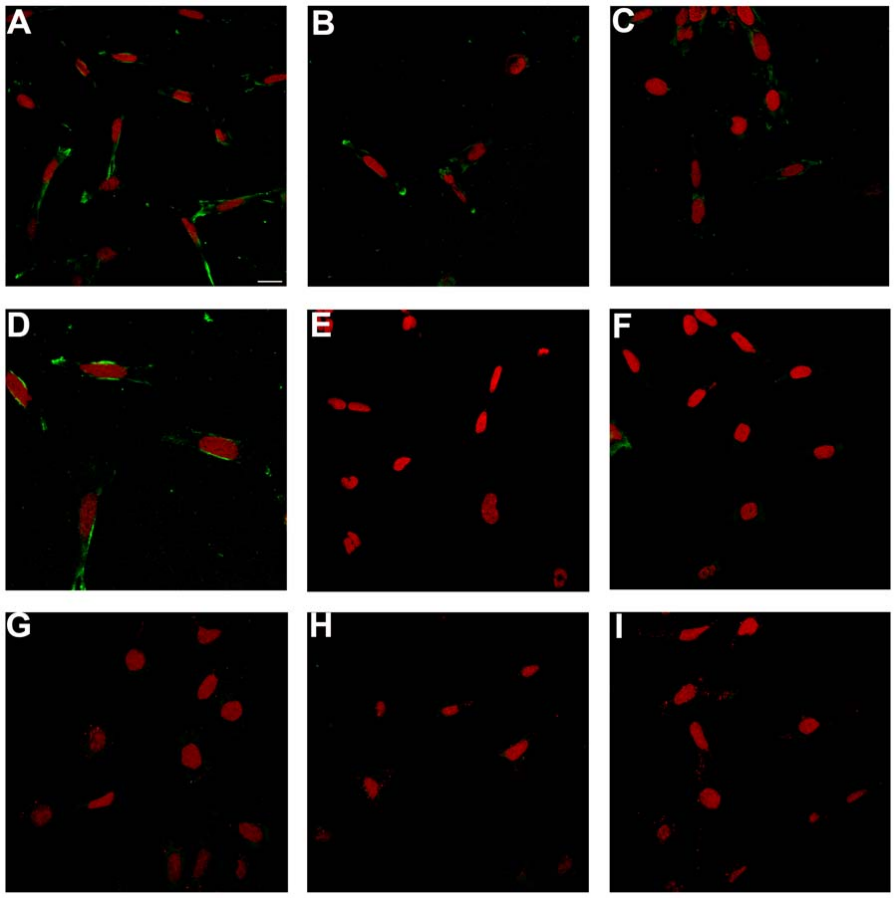
OPN5 Immunocytochemistry in RGC-5 cells (A to F)and HEK-293 cells (G to I). Immunostaining with anti-OPN5 antibody at dilutions 1∶600 (A and G), 1∶300 (B and H), and 1∶1000 (C and I). **D.** Optical zoom of a region of panel A. **E.** Negative control experiments without primary OPN-5 antibody in RGC-5 cells. **F.** Rat-β III Tubulin staining in non-permeabilized RGC-5 cells. Scale Bar: 20 µm. Each photomicrography is composed of single slices of merged images in which the green signal corresponds to OPN5 fluorescence and the red signal to propidium iodide (PI) fluorescence. See Materials and Methods for further detail.

**Figure 3:**
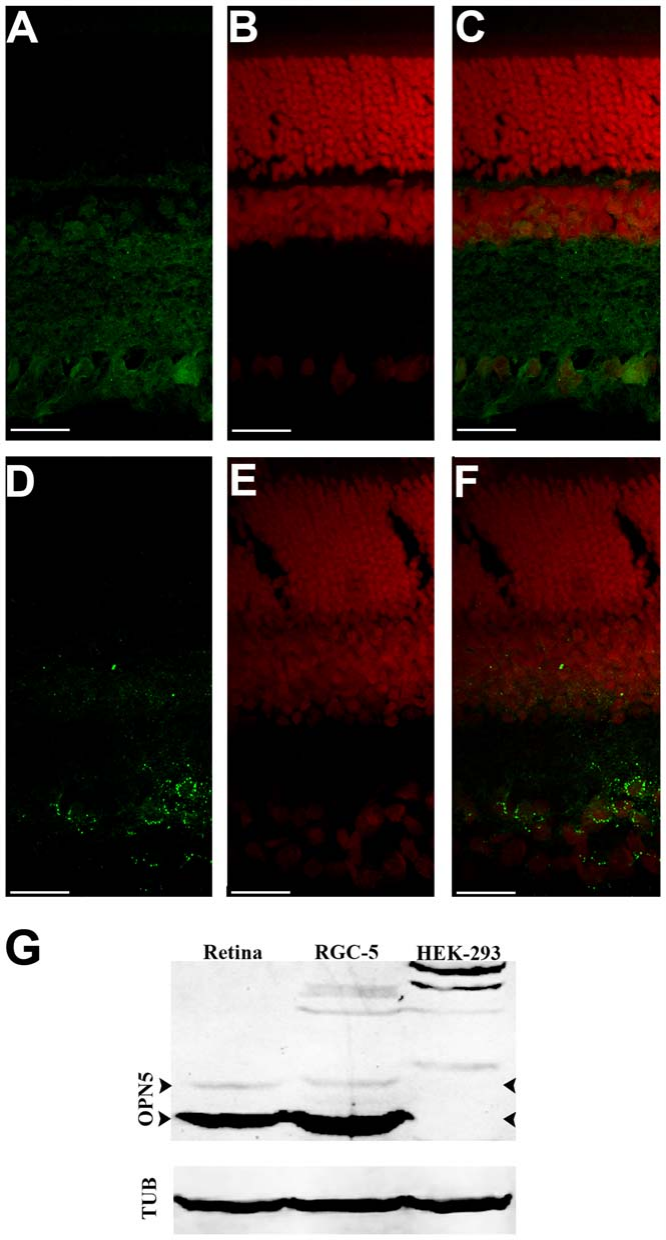
OPN5 Immunohistochemistry in rat retina and Western Blot Assay. **A and D.** Immunostaining of rat retina with anti-OPN5 antibody dilutions 1∶600 and 1∶1000 respectively. **B and E.** Propidium Iodide (PI) staining. **C and F.** Merge image of both OPN5 and PI fluorescence signals. Scale Bar: 20 µm. Each image is a 2D projection of a Z stack taken with a 60x objective. **G.** Western blot of homogenates of whole rat retinas, RGC-5 and HEK-293 cells using the anti-OPN5 antibody (dilution 1∶1000). α-Tubulin (Tub) was utilized as a housekeeping protein**.** The arrowheads demark bands of ∼40 KDa. See Results and Materials and Methods for further detail.

As OPN4 has been shown to be expressed in ipRGCs of different mammalian species, and since the RGC-5 cell line exhibits several RGC features, we examined the expression of this and other opsins in RGC-5 cells by RT-PCR. No traces of Opn4 or rhodopsin mRNAs were found after 40 RT-PCR cycles in RGC-5 cells as compared to positive controls with whole rat eye (Fig. 1A). We also investigated mRNA expression in non-visual opsins and/or photoisomerases such as *Opn3, Opn5* and *RGR* and the visual opsin, *Opn1mw* cone opsin. Detectable mRNA levels were seen in *Opn1mw*, *Opn3*, *Opn5* and *RGR* in RGC-5 cell cultures as observed in positive controls with whole rat eye (Fig. 1B). We assessed the presence of Opn5 protein in RGC-5 and HEK293 cells (Fig. 2) and in retinal sections of rat (Fig. 3) using immunochemistry with a specific polyclonal anti-Opn5 antiserum (dilution: 1∶300 to 1∶3000). Fig. 2 shows Opn5 (+) immunostaining in RGC-5 cultures at the different dilutions (Fig. 2 A-D). Immunofluorescence was found to be localized in the cellular membranes of non-permeabilized cells (see control with βIII-Tubulin in Fig. 2F). No detectable immunolabeling was observed in HEK293 cultures at the dilutions tested (Fig. 2 G-I). When Opn5 immunoreactivity was assessed in the retina of rat (Fig. 3 A-F), robust immunostaining was observed in the ganglion cell layer (GCL) at the different dilutions used (1∶600–1∶1000), demarcating cell somas and some processes (Fig. 3 A,C,D,F). Immunolabeling was also detected in the inner plexiform layer (IPL) and extending over a few cell somas of the inner nuclear layer (INL) (Fig. 3 A,C), especially at higher concentrations of the OPN5-antibody dilutions. Additionally, Fig. 3G shows a representative Western blot assay with homogenates of RGC-5 cells and rat retinas revealing two immunoreactive bands with the expected molecular weight (MW) for the rat OPN5 protein (∼40 KDa, arrowheads); strikingly, one of these bands was strongly immunostained while the other was weakly visualized. In RGC-5 extracts, other bands were slightly labeled at higher MWs probably related to OPN5 posttranslational modifications. The control cells HEK-293 do not present positive immunoreactivity for the bands observed in the rat retina; however, some immunolabeled bands of higher MW were visualized. Taking into account the ICC results (Fig 2 G-H) and the predicted MW of the human OPN5 protein (39–42 kDa) we speculate that the immunoreactivity seen in HEK-293 cells is likely associated to peptide epitopes exposed during the WB procedure.

Taking together, all these results are the first to show the expression of the OPN5 protein within the mammalian retina. Given the presence of OPN5-inmunoreactivity in the INL and GCL of the rat retina as well as in RGC-5 cells, these observations further support the inner retinal origin of this cell line.

### Light Responses of RGC-5 cells I: Differential Ca^2+^ Responses in RGC-5 cells exposed to Photic Stimulation

**Figure 4 pone-0026417-g004:**
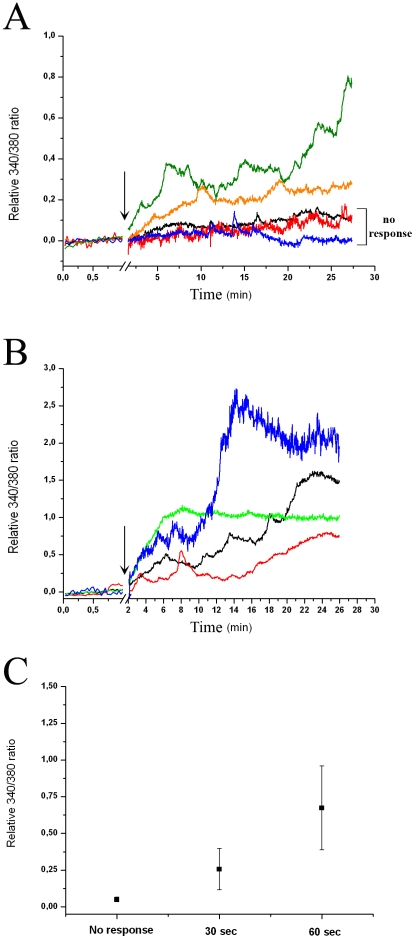
Intrinsic Photic Responses of RGC-5 cells by FURA-2AM Ca^2+^ Imaging. Relative 340**/**380 FURA-2AM ratio for individual cells showing differential Ca**^2+^** responses to brief unfiltered white light pulses of 20000 lux for 30 (**A**) or 60 (**B**) sec indicated by an arrow. **C**- Linear relationship between amplitude responses of A and B (5 min after the light stimulus ended) and stimulus duration.

Based on the presence of Opn5 and other visual and non-visual opsins (Fig. 1–2) and in order to investigate putative photic responses in RGC-5 cells, we assessed changes in intracellular Ca^2+^ levels in individual RGC-5 cells after light stimulation of different intensities (1000–20000 lux) and durations (30–60 sec). For this, we used the fluorescent indicator fura-2 AM and image analysis as previously described for the retina [43]–[45] and RGC-5 cells [29]. Fig. 4 shows the differential responses of RGC-5 cells to light exposure with white light of 20,000 lux for 30 (panel A) or 60 sec (panel B), in which each trace represents the calcium response of an individual cell. We found that some cells (28%; 2/7 cells) display an increase in their intracellular Ca^2+^ levels after the light stimulus (30 sec) whereas others exhibit no appreciable changes in Ca^2+^-related fluorescence levels. After 60 sec light stimulus all the tested cells (n = 4) exhibited significant changes in Ca^2+^-related fluorescence levels. Fig. 4 C shows the linear increase in the mean amplitude of photic responses to the duration of light stimulus as recorded 5 min after the termination of the stimulus.

**Figure 5:**
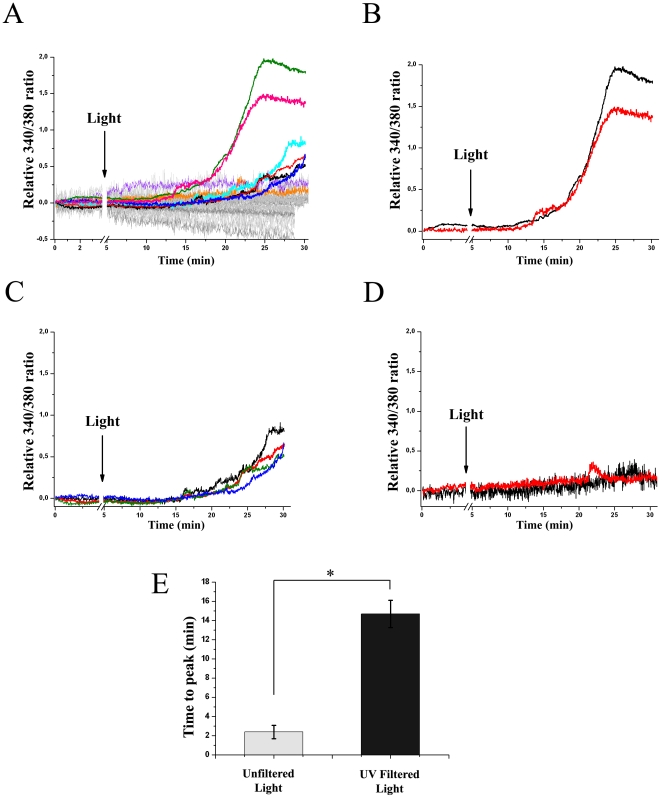
Intrinsic Photic Responses of RGC-5 cells by FURA-2AM Ca^2+^ Imaging. **A**. Relative 340**/**380 FURA-2AM ratio for individual cells showing differential Ca**^2+^** responses to brief UV filtered white light pulses of 20000 lux for 30 sec at the point marked with the arrow. Each gray trace represents the Ca^2+^ dynamics of a non-responsive single RGC-5 cell (n = 126). Colored traces represent the Ca^2+^ dynamics of responsive RGC-5 cells. Light responses in RGC-5 cells were sustained through time and are shown by amplitude level in separate plots: high (**B**), intermediate (**C**) and low (**D**). **E**. Time to peak responses of RGC-5 cells stimulated by a UV-unfiltered or UV- filtered white light pulse during 30 sec. Data mean ± SEM, p<0.05 by Student t-test.

When RGC-5 cultures were exposed to a 30 sec white light pulse by using filtered light (∼400 nm < λ <600 nm) of the same intensity (20,000 lux), we were able to measure significant fluorescent changes representing an increase in intracellular Ca^2+^ levels in around 5% (n =  8) (Fig. 5 A, colored lines) of cells tested (n = 134; Fig. 5 A). Strikingly, these responses were quite different from those obtained with unfiltered white light pulses since they showed a longer latency period (unfiltered light: 2.4±0.7 min vs. filtered-UV light: 14.7±1.4 min) as shown in Fig. 5 G.

In addition, cells treated with UV-filtered light pulses could be classified into high (Fig. 5 B), intermediate (Fig. 5 C) or low amplitude (Fig. 5 D) responsiveness over the time recorded (30 min). Additionally, at least one light-responding RGC-5 cell (n  = 1/18) was detected when cultures were exposed to brief pulses of filtered white light of lower intensity (1,000 lux) whereas NIH 3T3 cell cultures (utilized as non-retinal controls) showed no detectable light responses after exposure in any of the tested cells (n = 10) (data not shown).

### Light Responses of RGC-5 cells II: Photic Induction of c-Fos protein

**Figure 6:**
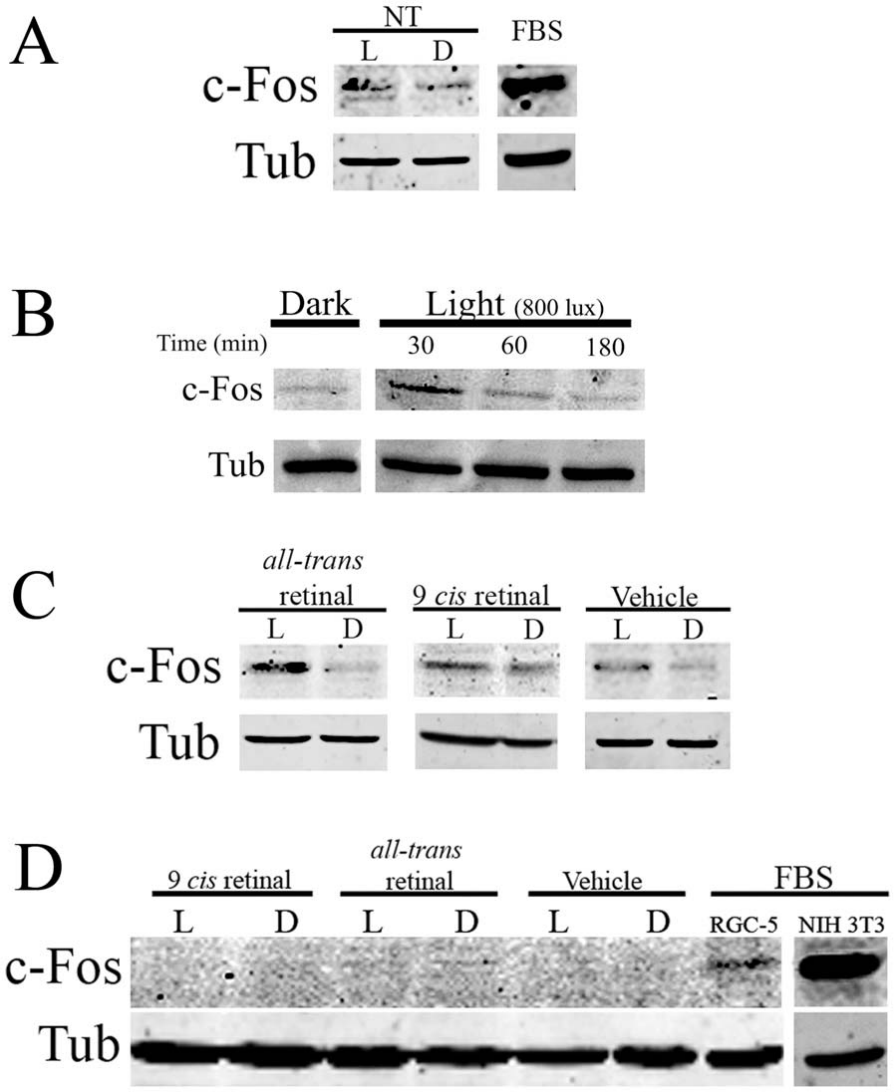
Induction of c-Fos protein in RGC-5 cells by light-stimulation. **A.** c-Fos induction in RGC-5 cells without retinoids treatment (NT) after a 30 min pulse of cool white light or controls kept in the dark (image representative of 2 independent experiments) **B.** c-Fos induction in Vitamin A-supplied RGC-5 cultures after 30, 60 and 180 min of light exposure (800–1000 lux) or controls kept in the dark by WB. α-Tubulin (Tub) was used as a housekeeping protein**. C.** Immunostaining for c-Fos protein in RGC-5 cells exposed to light or kept in dark in the presence of all-trans retinal, 9 cis retinal, vehicle (representative of at least 3 independent experiments) and positive control of c-Fos induction by FBS stimulation. **D**. Immunostaining for c-Fos protein in NIH 3T3 cells (negative controls) exposed to light or kept in dark in the presence of all-trans retinal, 9 cis retinal or vehicle or no treatment (NT), and a positive control of c-Fos induction by FBS stimulation in RGC-5 and NIH 3T3 cells (representative of 2 independent experiments).

RGCs of different vertebrate species have been shown to induce the expression of c-Fos and other IEG proteins in response to light [33]–[37], [46]–[48]. Here we investigated the induction of c-Fos protein in RGC-5 cells after exposure to white light of 800–1000 lux for 30 min compared to controls kept in the dark. As shown in Fig. 6A, slight LD differences were observed. Unlike in the controls kept in the dark, a 30 min light pulse was able to induce c-Fos (Fig. 6B) when light pulses of different durations (30, 60 and 180 min) were tested in the cell cultures in the presence of vitamin A. Levels of c-Fos immunoreactivity were higher at 30 min of light exposure and decreased at 60–180 min of light stimulation, resembling typical IEG behavior. In another series of experiments, cultures were incubated with different retinal isomers (all-trans retinal, 9 *cis* retinal) or vehicle. As shown in Fig. 6 C, significant LD differences in the induction of c-Fos protein were found in RGC-5 cell cultures incubated with all-trans retinal or vehicle. In those cultures treated with the isomer 9 *cis* retinal no clear LD differences were observed, although in some cases the dark levels of c-Fos were slightly higher than those of light-exposed cells (data not shown). NIH-3T3 cells were used as a negative control for light response specificity since these cells do not exhibit c-Fos induction by light under the same illumination condition used in RGC-5 cells (Fig. 6 D). The absence of light induction of c-Fos expression in NIH-3T3 cells does not indicate a lack of c-Fos responsiveness since serum-stimulation was able to induce this IEG expression in the cells (Fig 6 D and see [29]).

## Discussion

In mammals, both visual and non-visual photoreception is primarily restricted to the eyes and enucleation abolishes all responses to light [49], [50]. Novel photopigments have been recently described and potentially implicated in non-visual light responses. In this work, we show for the first time that the mammalian inner retina expresses the novel opsin OPN5. Further investigation will be required to elucidate its physiological relevance in mammals.

The RGC-5 cell line displays RGC-like features [29]–[31] and in previous work we demonstrated that the RGC-5 cell line expresses clock genes and displays the induction of c-Fos protein in response to serum stimulation [29]. In the current paper we report for the first time that RGC-5 cells exhibit characteristics associated with photosensitive cells: they express several visual and non-visual opsins/photoisomerases and display intrinsic photosensitivity, regulating levels of intracellular Ca^2+^ and c-Fos expression, akin to features associated with inner retinal cells [2], [4], [51].

### Expression of Opn5 and other visual and non-visual opsins

A wide range of opsins has been identified in vertebrates [2], [4], [52]–[55], all of which can be classified into fifteen gene families based on similarities in their aminoacidic sequence [4]. New opsin members (Opn3, Opn4, Opn5) have been identified over the last decade, but only one of them, Opn4 (melanopsin), has been extensively studied [1], [7], [8], [10], [13], [15], [56]. Opn4 was shown to be expressed in the ipRGCs involved in the setting of biological clocks, pupillary light reflexes and other non-visual functions [1], [2], [5]–[8], [10], [18], [50], [51], [56]. Based on the strong body of evidence showing Opn4 expression in ipRGCs and since it has been established that RGC-5 cells have an RGC origin [30], [31], we speculated that Opn4 could be present in RGC-5 cells. Although we did not find detectable levels of Opn4 (Fig. 1 A) or rhodopsin (this paper: Fig. 1A and [57]) mRNAs in this cell line, we did detect the presence of a cone opsin transcript (Figure 1 B). In agreement with our observations, recent reports have demonstrated the expression of cone opsins in cells of the inner retina, localized in the GCL of humans and rodents retinas [24] as well as in RGC-5 cells [57]. Strikingly, these cells also express the transcripts for a variety of putative photopigment/photoisomerases such as Opn5, Opn3 and RGR (Fig. 1B), most of which have as yet unknown functions in the eye.

RGR was initially identified from bovine retinal pigment epithelium (RPE) [58] and subsequently localized within the retina in RGCs [28] and/or Müller cells [59]–[65]. It seems more likely that RGR acts as a factor enhancing the classical visual cycle rather than a component of an independent photic visual cycle [65], [66]. However, whether RGR is a functional photopigment is still an open question.

Opn3 was originally designated as encephalopsin and then panopsin because it was found to be expressed within the retina and in a variety of extra-retinal tissues such as brain, testis, liver and lung [23], [26], [27], [67]. In the human retina, Opn3 protein is expressed in the different neural layers including the GCL [22]. Nevertheless, the function of OPN3 is still not known [22], [68], [69].

Given the widespread extra-retinal localization of Opn3 mRNA and its implication in immune responses, and the involvement of RGR in the regulation of the retinoid cycle, we focused on Opn5 (neuropsin) and carried out a more in-depth study at the mRNA and the protein level in the mammalian retina and RGC-5 cells (Fig. 1B, 2 and 3).

Opn5 mRNA was found to be expressed in mouse testis, brain and eye, as well as in human retina and brain [23]. However, prior to the present paper it was not known whether the Opn5 protein is expressed in mammalian retina and if so, what type of retinal cell population expresses this opsin.

Here we demonstrate that Opn5 mRNA is clearly expressed in rat eye (Fig. 1B) whereas OPN5 is specifically expressed within the retina, in INL and GCL cells and in IPL processes (Fig 3). Recent reports have shown that this opsin constitutes a functional UV-sensitive Gi-coupled bistable photopigment with maximal efficiency at ∼420 nm, probably involved in the photoreception necessary for seasonal reproduction in birds [21], [25]. In the chicken, it was shown to be expressed in the pineal gland and in neurons of the INL and GCL of the retina [21] reflecting a similar retinal distribution to that described in this work for the mammalian retina (Fig 3).

We can hypothesize that OPN5 expression in the retina of mammals is responsible for the detection of some remaining light such as observed in non-rod, non-cone, non-melanopsin animals (Gnat1-/- Cnga3-/- Opn4-/- mice) in which certain non-image forming tasks (a residual pupillary light reflex) are still observed [70], [71]. Additionally, retinal OPN5 could play a role in photoreception related to seasonal behavior in mammals since, unlike in birds, brain regions in adult mammals have not been shown to participate in light detection [72]. However, in rat neonatal pinealocytes the expression of both rod-specific and cone-specific phototransduction components has been reported [73], [74]. Also, Opn5 transcript was previously found in mouse brain, and it was suggested that this product may reflect Opn5 expression in the pineal gland in a similar manner to that described for rod and cone opsins [23]. Based on this body of evidence, it is therefore plausible that the mammalian pineal gland is physiologically photosensitive during early life, Opn5 being one of the elements involved in non-visual light detection [73], [74].

In this work, we also observed expression of both Opn5 mRNA and protein in RGC-5 cells (Fig. 1–3). Moreover, using confocal microscopy we detected that the expression of OPN5 in these cells was restricted to plasma membrane and positive inmunolabeling was absent in HEK293 cells as previously observed [21]. Since RGC-5 cells exhibited detectable light responses (Fig. 3–5), the presence of OPN5 and other opsins in these cells allows us to infer that at least one of them is functional and responsible for the responses described.

### Differential Ca^2+^ Responses to Light Stimulation in RGC-5 cells

One known feature of the photosensitivity in vertebrates for both PRCs and ipRGCs is the change in intracellular Ca^2+^ levels after light exposure, differentially causing cell hyperpolarization or depolarization respectively [3], [20], [43], [44], [75].

The RGC-5 cultures showed distinct and specific increases in Ca^+2^ responses to a brief white light stimulus (Fig. 4) whereas dark controls (bleaching controls) did not show this behavior (data not shown). As expected, shorter light pulses gave rise to a lower number of responsive cells than longer pulses (Fig. 4 A and B). This linear relationship was also observed between amplitude responses and stimulus duration (Fig. 4 C), which is a common feature in responses of Opn4-expressing ipRGCs [44]. In this respect, we used an illumination setting [43] in which no assessment of the Ca^+2^ dynamics was performed during the course of light stimulation. When we used filters to decrease the UV irradiation from light stimulus we found a lag of 10 min in the beginning of Ca^+2^ responses (Fig. 5). Under this light condition we only detected photic responses in 5% of the total RGC-5 cells measured (n =  134) which could be classified into three types: one exhibiting high and sustained responses for at least 10 min after onset of the response (Fig 5 B), cells showing intermediate responses (Fig 5 C) and cells with a low amplitude responsiveness that returned to basal levels after a few minutes (2–3 min, Fig 5 D). These results were unexpected since RGC-5 cells are a clonal cell line. However, as previously reported these distinct Ca^2+^ responses may reflect different subsets of RGC-5 cells based on morphology, Ca^2+^ responses to ATP and electrophysiology [29], [76] or may simply indicate insufficient chromophore availability in the media to be able to an adequate photic response in all cells. Given that OPN-5 positive immunoreactivity is present in many cells of our RGC-5 cultures and that only a subset of them displays Ca^2+^light responses, it is likely that those photosensitive cells also express Opn5. Nevertheless, the heterogeneous light responsiveness of RGC-5 cells may also suggest that some of these cells do not contain a full and/or functional phototransduction machinery, even when they would express the Opn5 photopigment. Clearly, Ca^2+^ responses observed in RGC-5 cells are totally different from those reported in ipRGCs since the RGC-5 responses are slower and sustained over time, a finding consistent with the absence of Opn4 expression in our cell line. It has been reported that no voltage-dependent inward Ca^2+^ currents are observed in RGC-5 cells [76]. However, these cells show Cl^-^ currents [76] and it has been suggested that chloride ions have greater relevance in intracellular signaling than previously thought [76], [77]. Moreover, some channels involved in Cl^-^ currents have been implicated in controlling intracellular Ca^2+^ signaling, likely facilitating Ca^2+^ release from the endoplasmic reticulum [78], [79]. In this context the higher intracellular Ca^2+^ levels observed in RGC-5 cells may involve the release of this cation from intracellular stores, thus possibly explaining the slower and sustained Ca^2+^ responses observed. However, as we were unable to measure Ca^2+^ levels throughout light stimulus, we cannot exclude some earlier Ca^2+^responsiveness.

A significant change in the profile of light responses was observed with the partial elimination of UV irradiation in the light stimulus: in the presence of UV-enriched light, RGC-5 responses began earlier, possibly indicating that a UV-sensitive photopigment is mediating the response (Fig. 5E). This observation correlates with the expression of OPN5 and RGR in RGC-5 cells since both opsins have a maximal absorbance in the UV-blue region of the spectra. However, we measured a remaining light responsiveness in RGC-5 cells exposed to UV-filtered light that could involve the absorption of OPN3 and/or cone opsins, both expressed in these cells.

The main aim of the present paper was to establish whether RGC-5 cells are intrinsically photosensitive. On the basis of the photic Ca^2+^ responses shown here, this could indeed be the case for at least a subpopulation of RGC-5 cells. To further support our working hypothesis, we determined the light induction of c-Fos protein in RGC-5 cell cultures.

### Induction of c-Fos in RGC-5 cells by Light Stimulation

One differential characteristic of RGCs in the retina of different vertebrate species is their ability to respond to the physiological stimuli of light by triggering the induction of IEG expression such as the c-Fos protein. Light induction of c-Fos in RGCs has been shown even in animal models of retinal degeneration suffering the absence of functional classical retinal PRCs (cones and rods); this strongly indicates that the photic responses observed correspond to an intrinsic capacity of these inner retinal neurons [35], [37]. RGC-5 cells in culture were also able to induce the expression of c-Fos in response to a light stimulus as shown in Fig 6. Together with the results of previous work from our laboratory that serum stimulation rapidly promotes the expression of c-Fos in RGC-5 cells [29], the present finding strongly suggests that these cells retain at least some characteristics of mature RGCs in terms of their endogenous responses to physiological signals promoting significant changes in IEG induction. Moreover, these cells require the presence of vitamin A derivatives to increase their response to light by inducing c-Fos expression. This observation agrees with earlier reports [13]–[15], [80]–[82] and with our previous findings in chicken RGCs [18] indicating that retinal derivatives increase light responses in cultured cells. We found that light was able to trigger a significantly higher response in RGC-5 cells treated with *all-trans retinal* (Fig 6 C) than in cells without retinal treatment (Fig 6 A). In addition, vehicle-treated RGC-5 cells showed a slight photic induction of c-Fos expression. The level of c-Fos induction in vehicle-treated cells was higher than that observed in non-treated cells but lower than that in cells treated with *all-trans* retinal. In this respect, vehicle solution is a BSA solution [81], [82] which likely promotes greater availability of basal levels of *all-trans* retinal present in the culture media and improves delivery to the cells [83]. However, when cells were pre-incubated with 9 cis retinal, responses varied from one experiment to another: in two out of three experiments, no significant LD differences were observed (Fig 6 C) whereas in the third, c-Fos levels in the dark were slightly higher than those in the light-exposed cultures (not shown). On the basis of the foregoing we may infer that responses in RGC-5 cells require an opsin that uses all-trans retinal as endogenous chromophore, though in view of the results obtained in the presence of 9 cis retinal, we cannot completely rule out the bistable nature of the photopigments present in the cultures. The photic induction of c-Fos in RGC-5 cells constitutes a specific light response, since NIH 3T3 cells exposed to identical light conditions exhibit no sign of c-Fos expression or detectable phototoxic effects as a consequence of the light induction of AP1-transcription factors (Fig D). We chose NIH3T3 cells as negative controls since it has been reported that UV-light can induce c-Fos in this non-photosensitive cell line as a consequence of phototoxic effects. However, the results obtained here discard this possibility [84], [85] as well as any possible toxic effect of the retinoid treatment on the cells [86]. Furthermore, it has been shown that apoptosis induced by light in RGC-5 cells is only achieved by constant light exposure (1000 lux) over longer periods (24–48 h) [87]. In this connection our observations also rule out the possibility that the photic induction of c-Fos in this cell line is a consequence of a deleterious effect of light.

### Conclusions

In summary, our findings show that RGC-5 cells have the necessary opsin machinery to render them intrinsically photosensitive: they express Opn5 mRNA and protein and a number of visual and non-visual photopigments with putative functional roles in the processes of light detection. Moreover, the presence of Opn5 protein in the inner retina of rat strongly suggests the possibility of a new type of photoreceptor cell in the mammalian retina. This is the first report clearly demonstrating that RGC-5 cultures specifically respond to light stimulation. This intrinsic capacity is typical of retinal cells in vertebrates and supports the notion that these cells retain the photosensitivity of photoreceptors such as ipRGCs and/or horizontal cells [19], [88].

## Materials and Methods

### Materials

All reagents were analytical grade. The specific antibody against rat c-Fos was raised in rabbit (Sigma Aldrich) and used in a dilution of 1∶500 for Inmunocytochemistry (ICC) and 1∶10,000 for Western Blot (WB). The α-tubulin (α-Tub) protein was detected by the mouse monoclonal DM1A antibody (Sigma-Aldrich) dilution 1∶1000 for ICC and WB. The OPN5 antibody was from NOVUS biologicals (Littleton, CO, 80160, USA) (Cat. number: NB110-74726). This antibody was developed in rabbit and used in dilutions from 1∶150 to 1∶3000. The secondary antibodies used for ICC were: Alexa Fluor 546 goat anti-mouse IgG; Alexa Fluor 488 goat anti-rabbit; and Alexa Fluor 488 goat anti-mouse IgG (dilution 1∶1000) from Invitrogen. The secondary antibodies used for WB were anti-rabbit IgG peroxidase conjugate and anti-goat IgG peroxidase conjugate from SIGMA (dilution 1∶1000); anti-rabbit IgG IRDye®800CW conjugated goat polyclonal and anti-mouse IgG IRDye®680CW conjugated goat polyclonal from Li-COR® IRDye® Infra Red Imaging Reagents (dilution 1∶25,000). The FluorSafe was from Calbiochem®. Propidium iodide (PI), retinoids, protease inhibitor, and other biochemical reagents were purchased from Sigma-Aldrich (USA). Vitamin A (Tanvimil) was from Raymos SAIC; Pluronic acid F-127 and Fura-2 AM were from Invitrogen.

### RGC-5 Cell Cultures, Light Exposure and Effector Treatment

The RGC-5 cell line was provided by Dr. N. Agarwal (North Texas Health Science Center, USA) at passage 13 on June 2005. RGC-5 cells were grown in 100-mm tissue culture plates in Dulbeccós modified Eagle medium (DMEM, Sigma-Aldrich), 10% FBS (Gibco), 200 U/mL of penicillin and 100 µg/mL of streptomicyn according to [30], and were incubated at 37°C with 5% CO_2_. Successive passages were made after 3–4 days in culture when cells achieved 80% of confluence. For selected experiments, cells were grown to 50–60% confluency, serum-deprived during 36 h, stimulated by 50% FBS (synchronization stimulus, zeitgeber time (ZT 0)) as reported [89], [90] and harvested every 30 min during 2 h after the serum shock to be used in control experiments of c-Fos induction. In another series of experiments retinoids were added to cells at ZT 16. After addition of vitamin A (20 µg/mL), all-trans retinal, 9cis retinal (5 µM) or vehicle (BSA solution) cells were kept in darkness during 1 h (ZT 17) and subsequently illuminated as previously reported [18] with cool white light (800–1000 lux) during 30 min or maintained in the dark (dark controls). The temperature of the cell incubator was carefully controlled with two thermometers and maintained constant at 37°C throughout the entire experiment. After light treatment, cells were harvested in PBS 1X and homogenized for Western blot assays. Retinoids were dissolved in a vehicle BSA solution according to [81], [82] and all procedures involving retinoids were performed in dim red light.

### Animal Handling, Immunohistochemistry (IHC) and Immunocytochemistry (ICC)

Animal handling was performed according to the *Guide to the Care and Use of Experimental Animals* published by the Canadian Council on Animal Care and approved by the local animal care committee (School of Chemistry, National University of Córdoba, Exp. 15-99-39796). Wistar rats (n = 3) were reared from hatching until day 30 on a 12 h:12 h light-dark (LD) cycle at a room temperature of 25°C with food and water *ad libitum*. Animals were sacrificed at postnatal day 30 (P30) and then enucleated. Eyes were rapidly removed, a small hole was made with a 25-gauge hypodermic needle at the level of the lens, and eyeballs were fixed overnight in 4% paraformaldehyde in buffered phosphate saline (PBS) at 4°C. They were then rinsed in cold PBS, transferred to a 30% sucrose solution for 2–4 days until they dropped to bottom of the tube and subsequently embedded in optimal cutting temperature resin (Tissue Tek). 6–8 µm-thick cryostat sections were then prepared and stored at −20°C until use. Immunostaining was performed as published previously [20]. Briefly, sections were permeabilized (5 min) and then saturated with PBS containing 3% BSA, 0.1% Tween, and 0.1% sodium azide for 30 min (Buffer A). Sections were incubated overnight at 4°C with anti OPN5 antibody at a final concentration ranging from 1∶150 to 1∶3000 in buffer A. After 5x washes (10 min each) with 1X PBS, the secondary fluorescent antibody was incubated for 1 h at room temperature after which propidium iodide staining was performed (1∶1000, 4 min, room temperature). Slides were washed thoroughly with PBS then mounted in Fluorsave and kept at 4°C overnight.

RGC-5 cells were grown to 30–50% confluence on 10 mm cover-slips, and then fixed with 3% paraformaldehyde-4% sucrose in PBS for 20 min at room temperature [29]. Non-permeabilized cells were blocked for 2 h with 1% BSA-PBS at room temperature and incubated overnight at 4°C with primary antibodies in PBS. They were then incubated with the corresponding secondary antibody for 1 h at room temperature, washed and mounted with Fluorsave.

The OPN5 ICC and IHC were visualized using an Olympus FV 300 spectral confocal microscope equipped with a 20x (NA: 0.75) and a 60x (NA:1.42) Uplan SApo oil-immersion objective. Fluorescence images were acquired using a 488 nm Argon or 543 nm Helium-Neon laser excitation line and emission was collected between 510–540 nm and 565–610 nm, respectively. In each independent experiment the PTM voltage, gain and offset were calibrated in accordance with the autofluorescence and secondary controls, with similar parameters for all experiments. The confocal aperture (CA) was set at 100 µm. Z-stacks were acquired and retinal image slices were used for making 2D projections with the Image J software using the maximum intensity projection method. In order to prove the membrane-like localization of OPN5 in RGC-5 cells, single slices are shown. Images are representative of at least two independent experiments with two technical repetitions each.

### Western Blotting (WB)

RGC-5 and NIH-3T3 cells were harvested in PBS buffer and lysed as previously reported [29]. Total protein content in the homogenates was determined by Bradford's methods. Cell homogenates were resuspended in sample buffer and heated at 90°C for 5 min. 50 µg of protein were separated by SDS-gel electrophoresis on 12%–15% polyacrylamide gels, transferred onto nitrocellulose membranes, blocked for 1 h at room temperature with 5% skimmed milk in PBS and then incubated overnight at 4°C with specific antibodies. Membranes were incubated with the corresponding secondary antibody in PBS during 1 h at room temperature followed by three washes with PBS for 15 min each. For Li-COR® IRDye® antibodies, membranes were scanned using an Odyssey IR Imager (LI-COR Biosciences). For peroxidase conjugate antibodies, membranes were developed with 4-Cl-Naftol/ H_2_O_2_ as substrate and scanned with an HP Photosmart C3180 scanner.

### RT-PCR assays

Total RNA was extracted from RGC-5 cells, rat retina, and muscle using TRIzol® reagent following manufactureŕs specifications (Invitrogen). The yield and purity of RNA were estimated by optical density at 260/280 nm. Following DNAse treatment (Promega), c-DNAs were synthesized from RNAs using MMLV reverse transcriptase polymerase (Promega) with oligo dT as primers according to the manufactureŕs specifications. The polymerase chain reaction was performed in a Labnet Multigen Thermal cycler using the GoTaq® DNA Polymerase (Promega). The PCR products were resolved in 1–2% agarose gel. Table 1 summarizes the primers employed (asterisks indicate the primer sequence obtained from the Harvard Primer Bank (mice) that differs in one (*) or two (**) nucleotides from the rat sequence).

**Table 1:** Conditions and Primers used in the RT-PCR experiments to assess different transcripts in RGC-5 cells.

gene	mRNA accession Number	Primer	Sequence 5′--->3′	Reference	Num. of cycles	Tm (°C)
*Rgr*	**Rattus Norvegicus NM_001107299.1**	**Forward**	GCTGGCTGTAGGAACAGTCC **	**Harvard primerBank** **ID 10946656a1**	**40**	**57**
		**Reverse**	**GCTGCAACAAGGGCATTCA**			
*Opn1mw*	**Rattus norvegicus NM_053548.1**	**Forward**	**TTGCTGACCTAGCAGAGACCA**	**Harvard primerBank** **ID 6679975a3**	**38**–**40**	**57**
		**Reverse**	AGCCTTCAATGACACACAGAG *			
*Opn5*	**Rattus norvegicus NM_181772**	**Forward**	**CTGGGGGACTATGCACCTGA**	**Harvard primerBank** **ID 32306526a2**	**38**–**40**	**57**
		**Reverse**	**TGAGAAAACAATCACAGCCGTT**			
*Opn3*	**Rattus norvegicus XM_573517.2**	**Forward**	**TCTCTACTCCAAGTTCCCGCG**	**Harvard primerBank** **ID 6753710a1**	**40**	**57**
		**Reverse**	**GAAGGTGACTCCGAACAGGG**			
*Opn4*	**Rattus norvegicus NM_138860.1**	**Forward**	**ATGTGGTGATCACACGTCCA**	**Sakamoto et al., 2004**	**40**	**57**–**60**
		**Reverse**	**TGATCCCAGGAGCAGGATGT**			
*Rhodopsin*	**Rattus norvegicus NM_033441.1**	**Forward**	**GCAGTGTTCATGTGGGATTG**	**Sakamoto et al., 2004**	**40**	**57**–**60**
		**Reverse**	**CTGCCTTCTGAGTGGTAGCC**			
*GAPDH*	**Rattus norvegicus NM_017008.3**	**Forward**	**AGACAGCCGCATCTTCTTGT**	**Nieto et al., 2010**	**32**–**40**	**57**–**60**
		**Reverse**	**TGATGGCAACAATGTCCACT**			

Asterisks indicate the primer sequence obtained from the Harvard Primer Bank (mice) that differs in one (*) or two (**) nucleotides from the corresponding rat sequence.

### Calcium Imaging by FURA-2 AM Fluorescence Microscopy

Calcium imaging was performed as previously reported [29]. Briefly, RGC-5 or NIH3T3 cells were grown in an 8-well Lab-Tek recording chamber (Nunc^TM^ , NY-USA) to 30–50% confluency and then incubated in a colorless DMEM (GIBCO) containing 0.1% of Pluronic acid F-127 and 5 µM fura-2 AM (Invitrogen) Ca^2+^ indicator dye for 30–40 min at room temperature. 12 bits- 4x4 binned fluorescence images for each wavelength were acquired and the quantification of fluorescence levels in the cells was carried out using the MetaMorph_ 4.5 software; the measurements of mean fluorescence intensity in each cell were background-corrected by subtracting the mean fluorescence of an area with no cells. Bleaching was corrected according to [91]. Nevertheless, no significant bleaching effects or changes in focus were detected during the acquisition. The Fura-2 ratios (R) were calculated as the ratio of the corrected mean fluorescence obtained for excitation at 340 nm to the corrected mean fluorescence obtained for excitation at 380 nm. The normalization of the ratios (relative 340/380 ratio) was done as R_t -_ R_0_ / R_0,_ where R_t_ is the ratio at a given time “t” and R_0_ is the mean of 40 pre-stimulus ratios. The baseline before light stimulation had a variability of 10–15%. Light-responding cells were defined as those exhibiting a reciprocal fluorescence change (340 vs. 380) and a variation in the fura-2 ratio greater than 20% from the baseline. This criterion has previously been used by other laboratories [43] to define light-responding ipRGCs in culture. The stimulating light consisted of broad-spectrum light from a 100W halogen bulb that passed through the condenser on the top of the microscope chamber. Light intensity was measured with a light detector (Datalogging Lightmeter, model 401036; Extech Instruments, Waltham, MA, USA) placed in the approximate plane of the microscope chamber. The intensity of light stimulus used was 20,000 lux for all experiments shown, even when the UV radiation from the stimuli was filtered with low- and high-pass filters (cut-off: 600 and 400 nm, respectively).

### Statistics

Statistical analyses involved the Student t-test and one-way analysis of variance (ANOVA) with Newman-Keuls post-hoc tests when appropriate.
